# Normal Saline solutions cause endothelial dysfunction through loss of membrane integrity, ATP release, and inflammatory responses mediated by P2X7R/p38 MAPK/MK2 signaling pathways

**DOI:** 10.1371/journal.pone.0220893

**Published:** 2019-08-14

**Authors:** Joyce Cheung-Flynn, Bret D. Alvis, Kyle M. Hocking, Christy M. Guth, Weifeng Luo, Reid McCallister, Kalyan Chadalavada, Monica Polcz, Padmini Komalavilas, Colleen M. Brophy

**Affiliations:** 1 Department of Surgery, Vanderbilt University Medical Center, Nashville, Tennessee, United States of America; 2 Department of Anesthesiology, Vanderbilt University Medical Center, Nashville, Tennessee, United States of America; 3 VA Tennessee Valley Healthcare System, Nashville, Tennessee, United States of America; Max Delbruck Centrum fur Molekulare Medizin Berlin Buch, GERMANY

## Abstract

Resuscitation with 0.9% Normal Saline (NS), a non-buffered acidic solution, leads to increased morbidity and mortality in the critically ill. The goal of this study was to determine the molecular mechanisms of endothelial injury after exposure to NS. The hypothesis of this investigation is that exposure of endothelium to NS would lead to loss of cell membrane integrity, resulting in release of ATP, activation of the purinergic receptor (P2X7R), and subsequent activation of stress activated signaling pathways and inflammation. Human saphenous vein endothelial cells (HSVEC) incubated in NS, but not buffered electrolyte solution (Plasma-Lyte, PL), exhibited abnormal morphology and increased release of lactate dehydrogenase (LDH), adenosine triphosphate (ATP), and decreased transendothelial resistance (TEER), suggesting loss of membrane integrity. Incubation of intact rat aorta (RA) or human saphenous vein in NS but not PL led to impaired endothelial-dependent relaxation which was ameliorated by apyrase (hydrolyzes ATP) or SB203580 (p38 MAPK inhibitor). Exposure of HSVEC to NS but not PL led to activation of p38 MAPK and its downstream substrate, MAPKAP kinase 2 (MK2). Treatment of HSVEC with exogenous ATP led to interleukin 1β (IL-1β) release and increased vascular cell adhesion molecule (VCAM) expression. Treatment of RA with IL-1β led to impaired endothelial relaxation. IL-1β treatment of HSVEC led to increases in p38 MAPK and MK2 phosphorylation, and increased levels of arginase II. Incubation of porcine saphenous vein (PSV) in PL with pH adjusted to 6.0 or less also led to impaired endothelial function, suggesting that the acidic nature of NS is what contributes to endothelial dysfunction. Volume overload resuscitation in a porcine model after hemorrhage with NS, but not PL, led to acidosis and impaired endothelial function. These data suggest that endothelial dysfunction caused by exposure to acidic, non-buffered NS is associated with loss of membrane integrity, release of ATP, and is modulated by P2X7R-mediated inflammatory responses.

## Introduction

The most common intervention in the hospital setting is the intravenous administration of fluid, and Normal Saline (0.9% NaCl, NS) is the most commonly utilized crystalloid solution for this purpose. NS does not contain a buffer system and has a pH that is acidic (5.0 +/- 0.2). A recent study demonstrated that the use of NS solution for resuscitation of the critically ill resulted in increased need for renal-replacement therapy, persistent renal dysfunction, and increased all-cause mortality when compared to balanced buffered electrolyte solutions [1, 2].

In addition to its use for intravenous fluid resuscitation, surgeons typically store human saphenous veins (HSV) in NS after harvest and before implantation as vascular bypass grafts [3]. It has been noted that storage of HSV in NS leads to impaired endothelial function [4–6], whereas storage in a buffered electrolyte solution (Plasma-Lyte, PL) preserves endothelial function [4]. Storage of HSV in PL in which the pH has been decreased to 5.0–6.0 leads to endothelial dysfunction, suggesting that it is the acidic nature of the solution that is injurious [4]. In spite of the mounting evidence that NS may be injurious to the endothelium and clinically detrimental, NS is still commonly used as a resuscitative and storage solution in hospital settings.

To better understand the mechanism of endothelial injury induced by NS, the effects of two of the most common crystalloid solutions (NS versus buffered PL) on endothelial function were compared in cultured human saphenous vein endothelial cells (HSVEC) as well as intact strips of rat aorta (RA), HSV, and porcine saphenous vein (PSV). A porcine hemorrhage and volume overload model [7] was utilized to determine the effect of resuscitation with NS compared to PL on systemic acidosis and endothelial function. We hypothesized that exposure of endothelium to NS would lead to loss of cell membrane integrity, resulting in release of ATP, activation of the purinergic receptor (P2X7R), and subsequent activation of stress activated signaling pathways and inflammation. Since endothelial function can be assessed by vascular smooth muscle relaxation responses as a function of nitric oxide (NO) release by the endothelium, physiologic function of intact vessels was used as a bioassay and NO signaling mechanisms were also examined.

## Materials and methods

### Materials

All chemicals were purchased from Sigma-Aldrich Co. (St. Louis, MO), unless otherwise indicated. SB 203580, and carbachol were obtained from EMD Millipore Corp. (Billerica, MA). TNFα, IFNγ and IL-1β were purchased from Gibco Life Technologies (Gaithersburg, MD). NS and PL were obtained from Baxter Healthcare Corporation (Deerfield, IL). Heparin was obtained from Hospira (Lake Forest, IL).

### Procurement of vascular tissues

Intact rat aorta (RA) was obtained from euthanized (CO_2_ exposure) 250-300g, female Sprague Dawley rats in accordance with the recommendations in the Guide for the Care and Use of Laboratory Animals of the National Institute of Health. The protocol was approved by the Institutional Animal Care and Use Committee of the Vanderbilt University Medical Center (Protocol Number: M01800194). Immediately after euthanasia (CO_2_ exposure), the thoracic RA was isolated via an incision along the mid-abdomen, placed in heparinized (10 Units/mL) PL, and transported to the laboratory for immediate testing. Porcine saphenous vein (PSV) was harvested from the lateral aspect of the leg from adult female Yorkshire pigs, 40-45kg (Oak Hill Genetics, Ewing, IL) under an approved Institutional Animal Care and Use Committee of the Vanderbilt University Medical Center protocol (Protocol Number: M01600009), placed in PL, and transported to the laboratory for immediate testing. Human saphenous veins (HSV) were collected from patients undergoing coronary artery bypass grafting after consent according to the protocol approved by the Institutional Review Board of the Vanderbilt University Medical Center (Protocol Number:090607). Segments were collected prior to any intraoperative manipulation, placed in PL, and transported to laboratory for immediate testing.

### Physiologic responses of intact vascular tissues

Vascular tissues were cut into rings (2–3 mm), treated as described, and suspended in a muscle bath. At least 2 rings are used for each condition. Tissues were equilibrated in bicarbonate buffer (120 mM sodium chloride, 4.7 mM potassium chloride, 1.0 mM magnesium sulfate, 1.0 mM monosodium phosphate, 10 mM glucose, 1.5 mM calcium chloride, and 25 mM sodium bicarbonate, pH 7.4), equilibrated with 95% O_2_ / 5% CO_2_ at 37°C for 1 hour. Rings were manually stretched to 3 grams of tension, followed by a resting tension of 1 gram for an additional hour to produce maximal force-tension relationships as described previously [8]. After equilibration, rings were contracted with 110 mM potassium chloride (KCl) to determine functional viability. The tissues were then pre-contracted with phenylephrine (PE, 5 X 10^−7^ M) and relaxed with carbachol (CCH, 5 X 10^−7^ M), an acetylcholine analogue, to determine endothelial-dependent relaxation responses [9]. Force measurements were obtained using the Radnoti force transducer (model 159901A, Radnoti LLC, Monrovia, CA) interfaced with a PowerLab data acquisition system and LabChart software (AD Instruments Inc., Colorado Springs, CO). Relaxation was calculated as percent change in stress compared to the maximal PE-induced contraction (set as 100%).

To determine the effect of incubation in clinically relevant solutions, tissues were incubated in heparinized (10U/ml) PL or heparinized NS in the absence or presence of apyrase (Apy; 4U/ml); P2X7R inhibitors [oxidized ATP (oATP; 100μM), A740003(A74;100μM) [10], A438079 (A43; 100μM), brilliant blue FCF (FCF; 100μM)], P2X7R agonist 2’(3’)-O-(Benzoylbenzoyl) adenosine 5’–triphosphate (BzATP; 500μM), or p38MAPK kinase inhibitor SB203580 (SB; 20μM), for 2 hours.

To determine the effects of IL-1β on endothelial-dependent relaxation, IL-1β (10, 50, and 100ng/ml) in the absence or presence of SB (20μM) was added to the muscle bath and RA were incubated for 3 hours.

### Culture of primary human saphenous vein endothelial cells (HSVEC)

Primary HSV endothelial cells (HSVEC, PromoCell, Heidelberg, Germany) were grown in Endothelial Cell Growth Medium (PromoCell), maintained in a 37°C and 5% CO_2_ incubator, and passaged at 80% confluence. Early passage cells (between passages 2 and 6) were used in the experiments. The pH of NS and PL was determined by measuring the pH of different lots (n = 5) of each solution with a pH meter (AB15, Accumet, Fischer Scientific, Hampton, NH) after calibration with Symphony electrode solutions (Orion, Thermo Scientific, Waltham MA).

### Membrane integrity of HSVEC

HSVEC were untreated or incubated in NS or PL for 2 hours (to recapitulate the time the vein segment is stored in the OR after removal and before implantation as an autologous transplant into the arterial circulation). To determine the effect of NS on HSVEC morphology, the cells were fixed and stained with phalloidin to visualize actin and DAPI to visualize nuclei. To determine the effect of NS incubation on membrane integrity, medium was collected for measurement of lactate dehydrogenase activity (LDH, CytoTox-ONE), according to manufacturer’s protocol (Promega, Madison, WI) and ATP release as described previously using the ATP Bioluminescence Assay Kit (FL-AAM; Sigma, St Louis, MO) [11]. Transendothelial cell electric resistance (TEER) was measured with an epithelial voltohmmeter (World Precision Instruments, Sarasota, FL), using STX2 chopsticks electrodes (World Precision Instruments). HSVEC (passages 3–6) were plated (75,000 cells) on the polycarbonate membrane (0.4 μm) in 12 mm transwell inserts (12 well tissue culture plate, Costar, Corning, NY) coated with a solution of human fibronectin (50μg/ml in phosphate buffered saline; Sigma, St Louis, MO). The cells were cultured until TEER was ≥20 Ω.cm (a voltage that represents an intact endothelial monolayer) and treated with NS or PL for 2 hours and changes in TEER was measured. NS was changed to control medium after 2 hour of incubation to prevent cells from detaching from transwell inserts. TEER was also measured at 0.5, 2, 24, 48 and 72 hours after NS or PL removal at 2 hours.

### Inflammatory responses in HSVEC

Primed HSVEC (10 ng/ml TNFα & 100 ng/ml IFNγ for 48 hours) were treated with ATP (3mM) in the absence or presence of A43 (50μM), FCF (100μM), oATP (100μM), or SB (10μM) for 2 hours. Supernatants were collected and concentrated. IL-1β was measured using the Human IL-1β ELISA high sensitivity kit (eBioscience, San Diego, CA). To determine VCAM protein level, HSVEC were treated with ATP (1mM) in the presence or absence of A43 for 6 hours and harvested for immunoblotting.

### Immunoblotting

To determine the signaling pathways activated by NS and extracellular ATP injury, cells or tissues were harvested after treatment, proteins extracted using lysis buffer containing 50 mM Tris. Cl pH 7.4, 140 mM NaCl, 1% NP40, 1 mM EDTA, 1 mM EGTA, 0.5% deoxycholic acid with protease and phosphatase inhibitor cocktail, and separated by SDS polyacrylamide gel electrophoresis. Proteins were transferred to nitrocellulose membranes and immunoblotted with antibodies specific for phosphorylated and non-phosphorylated p38 MAPK, phosphorylated and non-phosphorylated MK2 (Cell Signaling Technology), vascular cell adhesion molecule (VCAM) (Santa Cruz, CA), endothelial nitric oxide synthase (eNOS), phosphorylated eNOS, arginase II (Cell Signaling Technology), and GAPDH (Millipore, MA). Protein-antibody complexes were visualized and quantified using the Odyssey Infrared Imaging System (LiCor Biosciences, Lincoln, NE). Protein levels were normalized to GAPDH level. Phosphorylation was calculated as a ratio of the phosphorylated protein to total protein.

### Porcine hemorrhage/volume overload resuscitation model

Under the approval of the Institutional Animal Care and Use Committee protocol (M1700129), adult female Yorkshire pigs (n = 6), 40-45kg (Oak Hill Genetics, Ewing, IL), were subjected to a hemorrhage and resuscitation protocol similar to that described previously [7]. General anesthesia was induced with telazol (4.4 mg/kg) ketamine (2.2 mg/kg), and xylazine (2.2 mg/kg) IM administration, and maintained with inhaled 2% isoflurane/1% oxygen. The animals were intubated and ventilated with a volume-controlled ventilator [(Hallowell EMC, Pittsfield, MA), 8mL/kg tidal volumes with a positive end-expiratory pressure of 5 cmH_2_O, I:E ratio 1:2] to maintain an end-tidal CO_2_ of 35 to 40 mm Hg. Intravenous heparin was administered (10,000IU bolus and 5000IU every 2 hours). The left carotid artery was cannulated with a 14g angiocatheter (MILA International, Florence, KY) for use in arterial pressure measurements and obtaining serial blood gasses. Surgical exposure of both internal jugular veins and cannulation with an 8.5 F catheter was performed for blood removal and placement of a Swan Ganz Catheter (PAC) (Edwards Lifesciences, Irvine, CA). Baseline hemodynamic measurements were obtained, and venous blood was removed by gravity, into a sterile empty intravenous fluid bag that was placed on the floor, in 50 mL increments until 400 mL of blood was removed or the MAP decreased < 40 mm Hg. 30 minutes following the hemorrhage protocol, the autologous blood was returned at a continuous rate of 50 mL/minute. The pigs were randomized to receive resuscitation with either NS or PL, maintained at 40° C. Resuscitation was delivered in 500 mL boluses and hemodynamic measurements were obtained after each infusion. Resuscitation was stopped when the cardiac output decreased (over the apex of the “Starling curve”). Pulmonary capillary wedge pressures (PCWP) were obtained after balloon inflation at end-expiration with the transducer height at the level of the right atrium. Central venous pressures (CVP) were obtained from pressure transducing the proximal port of the PAC. Arterial samples of heparinized blood were analyzed with an ABL5 blood gas analytical device (Radiometer, Copenhagen, Denmark). At the end of the hemorrhage/resuscitation study protocol, PSV were harvested prior to euthanasia and tested for endothelial-dependent relaxation in the muscle bath.

### Statistical analyses

Data are reported as mean ± standard error of the mean. Statistical significance (p <0.05) was determined using GraphPad Prism version 7.0. Paired t-tests were conducted for paired samples (samples treated from same tissues or cell passage. Unpaired t-test, One-way ANOVA analyses with Tukey’s Multiple Comparison test between groups was used to compare resuscitation treatments on different pigs. Linear regressions were used to determine correlation (r-value) between hemodynamic parameters and volume lost throughout hemorrhage and given during resuscitation. Significant changes in blood pH, ABE, and bicarbonate were determined using a Friedman non-parametric two-way ANOVA comparing gas levels prior to hemorrhage to gas levels at each point of interest. Dunnett’s Multiple Comparison Test was used to correct for multiple comparisons and is reflected in reported p-values for parametric datasets.

## Results

### Incubation of human saphenous vein endothelial cells in NS but not PL leads to loss of membrane integrity

**Fig 1:**
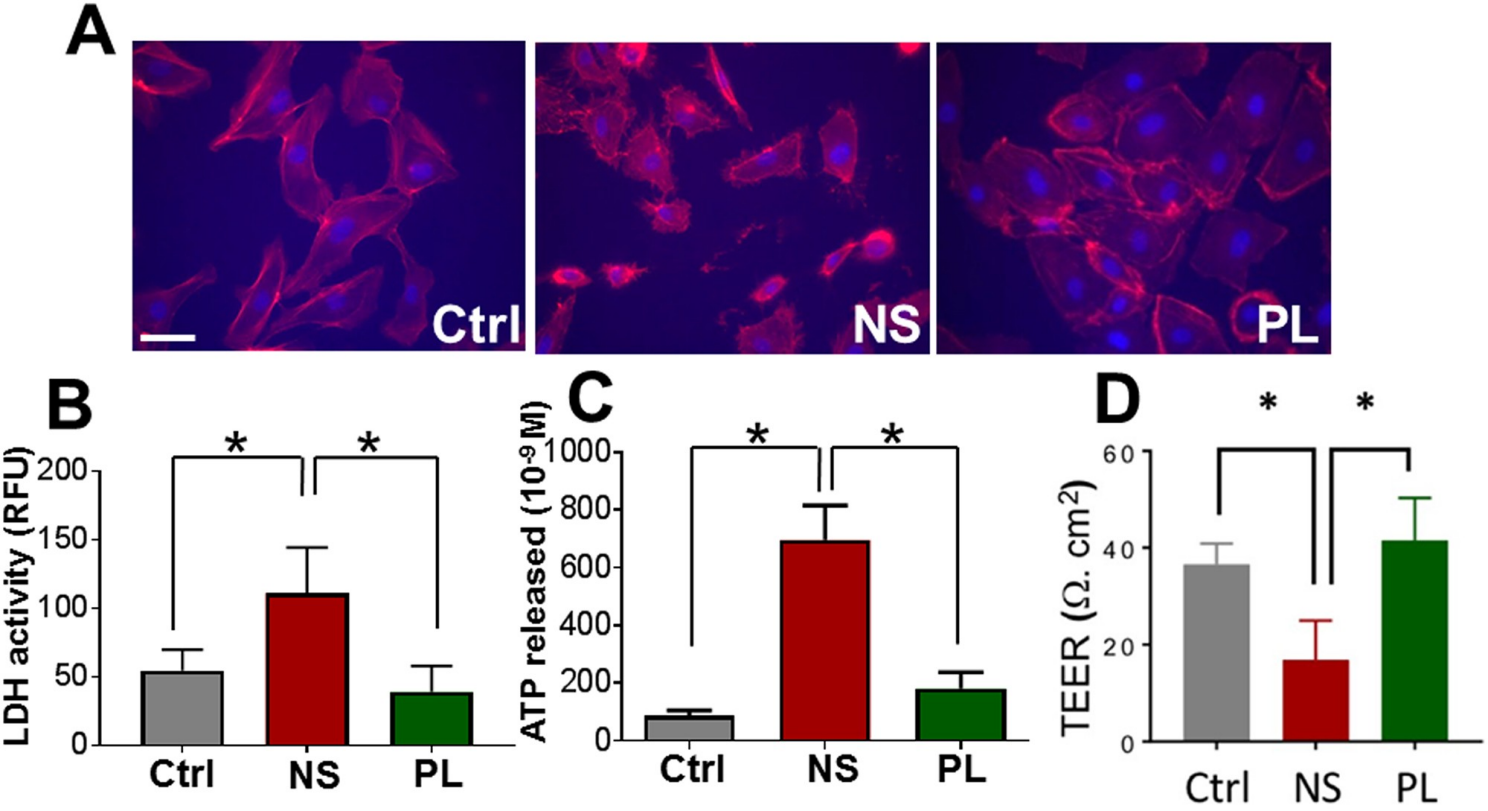
Incubation of human saphenous vein endothelial cells (HSVEC) in Normal Saline but not Plasma-Lyte leads to loss of membrane integrity. HSVEC were incubated in basal medium (Ctrl), Normal Saline (NS) or Plasma-Lyte (PL) for 2 hours, fixed, and stained with phalloidin (**A**, red stain; scale bar = 5μm). Supernatants were collected and LDH activity (**B**) and ATP levels (**C**) were measured (*p < 0.05, n = 3–5, where n = the number of cell plates from different passages of cells). Transendothelial electrical resistance (TEER) (**D**) was measured 2 hours after treatment with NS with an epithelial voltohmmeter using STX2 chopstick electrodes (*p < 0.05, number of cell plates (n) = 6 in triplicates from different passages of cells).

Experiments were first performed to determine the effect of NS compared to PL on endothelial cells. Human saphenous vein endothelial cells (HSVEC) incubated in NS, but not PL, exhibited abnormal rounded morphology (Fig 1A). Incubation in NS led to an increase in LDH activity [54.6 ±7.7 RFU, (control, Ctrl) vs. 111.0 ±19.2 RFU, (NS) and 39.1 ±8.4 RFU, (PL), p< 0.05, Fig 1B] and increased extracellular ATP concentration [696.9 ±119.3x10^−9^M (NS) vs. 87.2 ±18.3 x 10^−9^ M, (control, Ctrl) and 182.0 ±55.7 x 10^−9^M, (PL)], p<0.05, Fig 1C) in the medium of HSVEC.

To determine if impaired endothelial function was a consequence of alteration in membrane integrity due to NS exposure, Plates of HSVEC were treated with NS or PL and transendothelial electrical resistance (TEER) was determined (Fig 1D, S1 Fig). HSVEC incubated in NS, but not PL, had significant decrease in TEER at 2 hours [36.5 ± 1.8 Ω.cm^2^ (ctrl) vs 16.8.0 ± 3.3 Ω.cm^2^ (NS) vs 41.5±3.6 Ω.cm^2^ (PL), p < 0.05].

### Incubation of intact vascular tissues in NS leads to impaired endothelial-dependent relaxation

**Fig 2:**
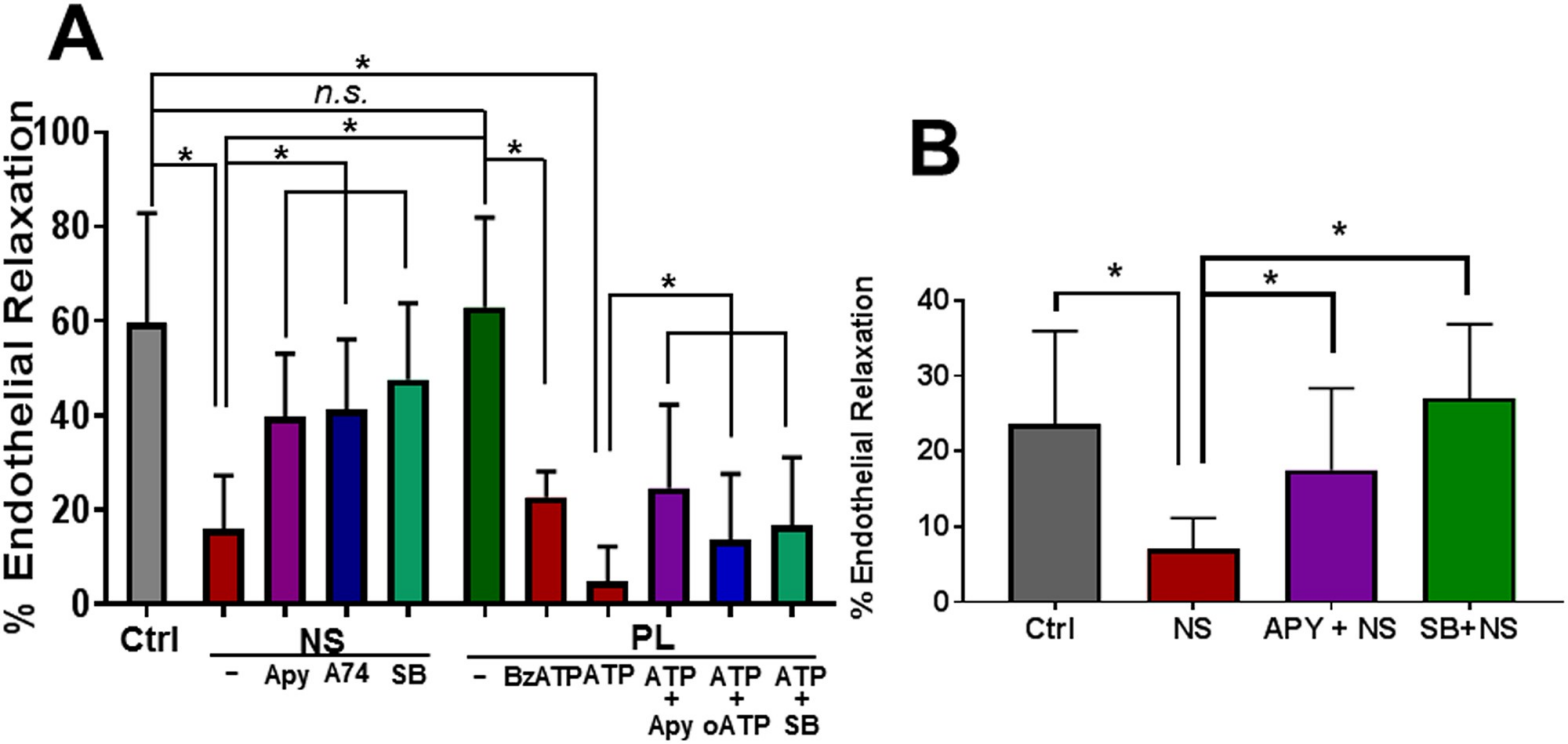
Incubation in Normal Saline solution but not Plasma-Lyte leads to impaired endothelial dependent relaxation of rat aorta and human saphenous vein. Rings of rat aorta (RA, n = 5–17 rats) were suspended in the muscle bath (Ctrl) or incubated for 2 hours in NS or PL in the presence or absence of 2’(3’)-O-(Benzoylbenzoyl) adenosine 5’–triphosphate (BzATP, 500μM); ATP (20mM), apyrase (Apy, 4 units/ml), A740003 (A74, 100 μM), or SB203580 (SB, 20 μM) (**A**). Rings of human saphenous vein (HSV, n = 5–6 patients) were suspended in the muscle bath (Ctrl) or incubated for 2 hours in NS in the presence or absence of apyrase (Apy, 4 units/ml) or SB203580 (SB, 20 μM) (**B**). The strips were suspended in a muscle bath and pre-contracted with phenylephrine (5 x 10^−7^M) and treated with carbachol (5 x 10^−7^ M), *p < 0.05.

To determine the physiologic relevance of the findings with HSVEC, intact strips of RA were incubated in NS and PL and suspended in a muscle bath to determine endothelial-dependent relaxation to CCh after PE-induced contraction. Exposure to NS but not PL led to impaired endothelial-dependent relaxation [15.9±4.6% (NS) vs 62.9 ±7.8% (PL), p < 0.05, Fig 2A], compared to control untreated RA [59.5 ±5.6%, Fig 2A]. When RA was incubated in NS in the presence of apyrase, an enzyme that hydrolyzes ATP (4U/ml), endothelial function was restored [39.9 ±5.4% (Apy), Fig 2A]. Treatment with the P2X7R inhibitor, A740003, or the p38 MAPK inhibitor, SB203580, also ameliorated endothelial dysfunction [41.2 ±7.5% (A74), or 47.6 ±6.1% (SB) vs 15.9 ±4.6% (NS), p < 0.05, Fig 2A].

To determine if the NS-induced endothelial dysfunction is associated with release of exogenous ATP, RA was incubated in PL in the presence of ATP or BzATP, a stable analogue of ATP. Both ATP and BzATP led to impaired endothelial-dependent relaxation [22.7±2.1% (BzATP) and 4.9 ±2.2% (ATP) vs 62.3 ±4.3% for control, p< 0.05, Fig 2A]. Co-treatment with BzATP and apyrase, oATP or SB203580 ameliorated endothelial dysfunction (Fig 2A).

Treatment of another vascular tissue, human saphenous vein (HSV), with NS but not PL led to impaired endothelial dependent relaxation [7.0 ±4.1% (NS) and 23.6±12.4% (PL), Fig 2B]. Treatment with apyrase or SB203580 ameliorated endothelial dysfunction associated with incubation in NS [17.5± 10.8% for (APY+NS) and 27.0± 9.7% (SB+NS), Fig 2B].

### Signaling events activated by NS injury

**Fig 3:**
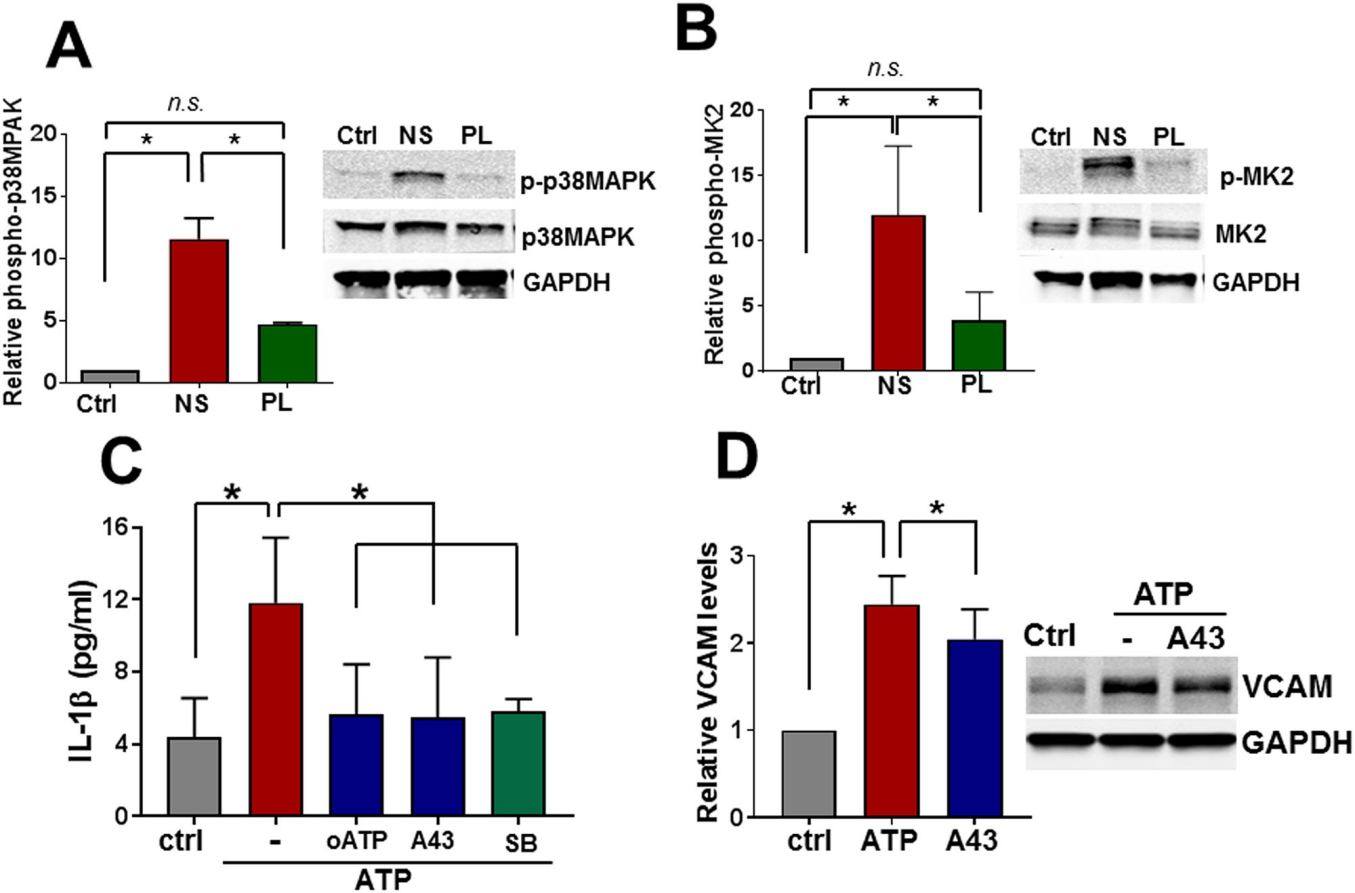
Stimulation of human saphenous vein endothelial cells (HSVEC) leads to activation of p38 MAPK/MAPKAP kinase 2; IL-1β release; and increased VCAM expression. HSVEC (n = 4–5 duplicate plates from different passages of cells) were incubated in growth medium (Ctrl), Normal Saline (NS) or Plasma-Lyte (PL) for 2 hours. The cells were lysed, lysates separated and immunoblotted for activated (phosphorylated, p-p38 MAPK) and total p38MAPK (**A**) or activated (phosphorylated, p-MK2) and total MK2 (**B**). Representative immunoblot and cumulative data of relative phosphorylation compared to untreated cells are shown (*p < 0.05). For IL-1β expression, HSVEC were primed with TNF (10ng/ml) and IFN (100ng/ml) for 48 hours and then treated with ATP (3mM) for 2 hours in the presence or absence of oATP (300μM), A438079 (A43; 50μM), or SB203580 (SB, 10μM), or MK2 inhibitor (MK2i, 100μM) for 24 hours and IL-1β was measured in the medium using an ELISA kit (**C**, *p < 0.05, n = 5–6). Cells were treated with ATP (1mM) for 6 hours in the absence or presence of A43 (50μM) and lysates prepared and immunoblotted for VCAM levels (**D**, *p < 0.05, n = 5–6 plates of cells from different passages).

Incubation of HSVEC in NS but not PL led to increases in the phosphorylation (activation) of p38 MAPK [11.6 ±1. 7-fold (NS) vs 4.7 ±0.14 (PL), p < 0.05, Fig 3A]. One of the downstream substrates of P38 MAP kinase is MAPKAP kinase 2 (MK2) which stabilizes cytokine mRNA and activates inflammatory pathways [12–15]. Incubation of HSVEC in NS but not PL led to increases in MK2 phosphorylation [12.0 ±3. 2-fold (NS) vs 4.0 ±1.07 fold (PL), p < 0.05, Fig 3B].

P2X7R activation leads to release of the pro-inflammatory cytokine, IL-1β in inflammatory cells (macrophages and monocytes) [16], neuronal cells [17], and human umbilical vein endothelial cells. MK2 signaling promotes production of pro-inflammatory cytokines including interleukin 1β (IL-1β; [18, 19]. To determine whether activation of endothelial P2X7R results in IL-1β release, primed HSVEC were treated with ATP. Levels of IL-1β release were elevated after ATP treatment and this increase was reduced by co-treatment with inhibitors of P2X7R (A43, oATP) and p38MAPK (SB) (Fig 3C).

Treatment of HSVEC with exogenous ATP led to increased VCAM expression which was reduced in the presence of the P2X7R antagonist A438079 (A43; Fig 3D), suggesting that exogenous ATP activates an inflammatory response in HSVEC.

### IL-1β-treatment leads to endothelial dysfunction

**Fig 4:**
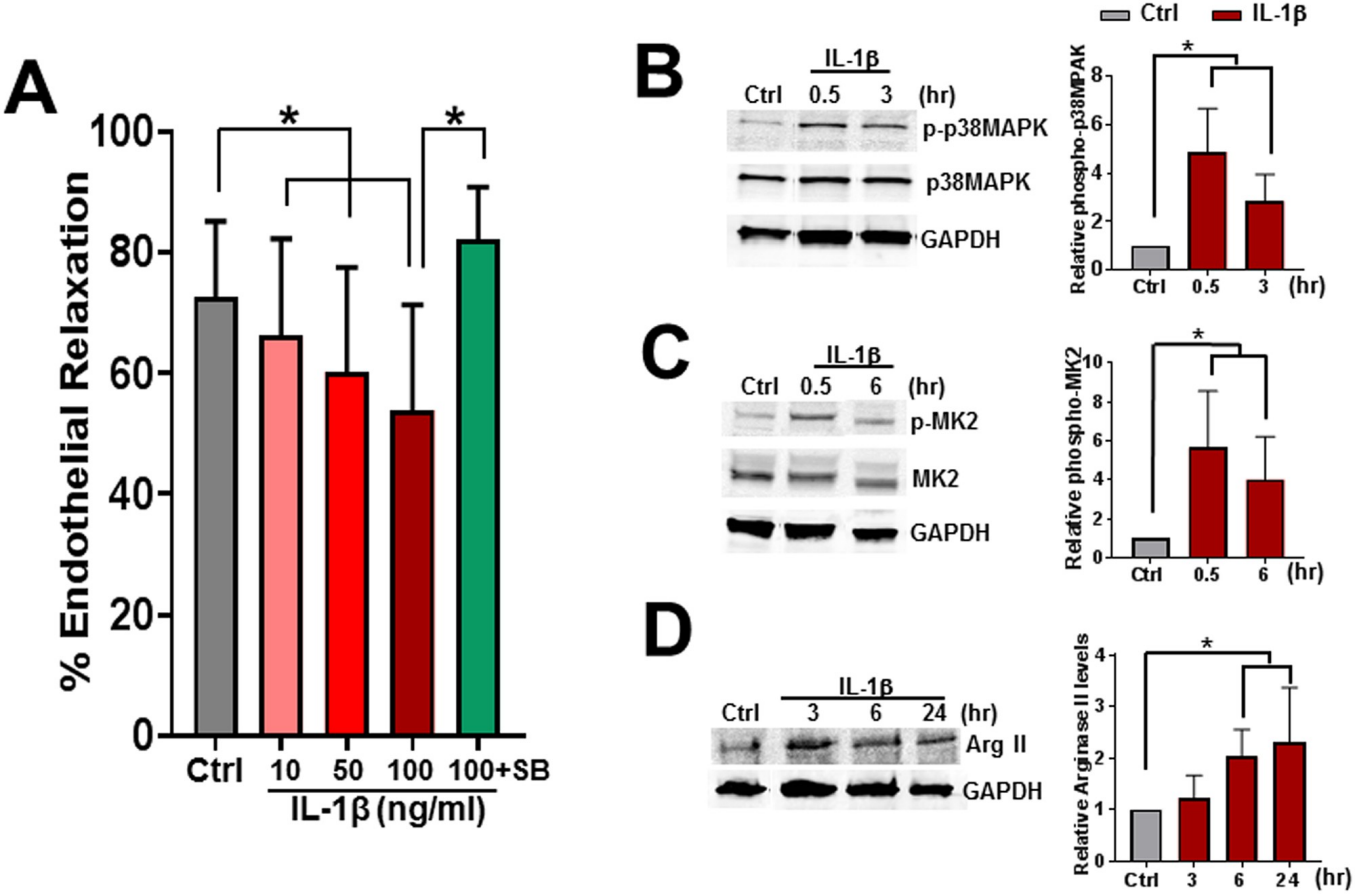
Treatment with IL-1β impairs endothelial-dependent relaxation in rat aorta (RA), activates p38MAPK and MK2 and increases arginase II activity in HSVEC. (**A**) Rings of RA (n = 12–20 rats) were suspended in the muscle bath and left untreated (Ctrl), treated with IL-1β (10, 50, and 100ng/ml), or co-treated with IL-1β (100ng/ml) and SB203580 (20μM) for 3 hours. Tissues were pre-contracted with phenylephrine (5x10^−7^M) and treated with carbachol (5x10^-7^ M) and endothelial relaxation was measured, *p < 0.05, n = 12–20 rats. HSVEC were either untreated (Ctrl) or treated with IL1-β (10 ng/ml) for the times indicated. Cells were lysed, separated and immunoblots were probed for activated (phosphorylated, p-p38MAPK) or total p38MAPK (**B**), activated (phosphorylated p-MK2) or total MK2 (**C**), and arginase II (ArgII, **D**). Representative immunoblots and cumulative data of relative phosphorylation compared to untreated cells shown. *p < 0.05, n = 4–5.

Since treatment of HSVEC led to increase in IL-1β, we next determined the effect of treatment with IL-1β on endothelial function in RA. IL-1β dose-dependently impaired endothelial relaxation of PE pre-contracted RA after 3 hours of treatment (Fig 4A). The effect of IL-1β on endothelial dysfunction was prevented by co-treatment with SB (Fig 4A) suggesting that IL-1β signals via a p38MAPK-associated pathway to impair endothelial function. p38MAPK is activated in response to inflammatory cytokines and the p38MAPK/MK2 signaling pathway also regulates inflammatory responses [20]. In HSVEC treated with IL-1β, immunoblotting experiments demonstrated that IL-1β led to increased p38MAPK and MK2 phosphorylation in a time-dependent manner (Fig 4B and 4C). IL-1β treatment led to rapid phosphorylation of p38MAPK and MK2 at 30 minutes, and phosphorylation of p38MAPK and MK2 remained elevated for up to 3 and 6 hours (S2 Fig), respectively.

Previous findings in our laboratory suggested that treatment of HSVEC with ATP, released after NS injury, reduces NO production [21]. NO production is regulated by synthesis (via activated eNOS) or substrate availability (via arginase). eNOS expression and phosphorylation were not altered by IL-1β treatment (S3 and S4 Figs). However, treatment with IL-1β led to increased levels of arginase-II (Fig 4D), suggesting that impaired relaxation may be due to decreases in NO resulting from decreased substrate availability.

### Volume overload resuscitation with Normal Saline but not Plasma-Lyte leads to systemic acidosis and endothelial dysfunction

**Fig 5:**
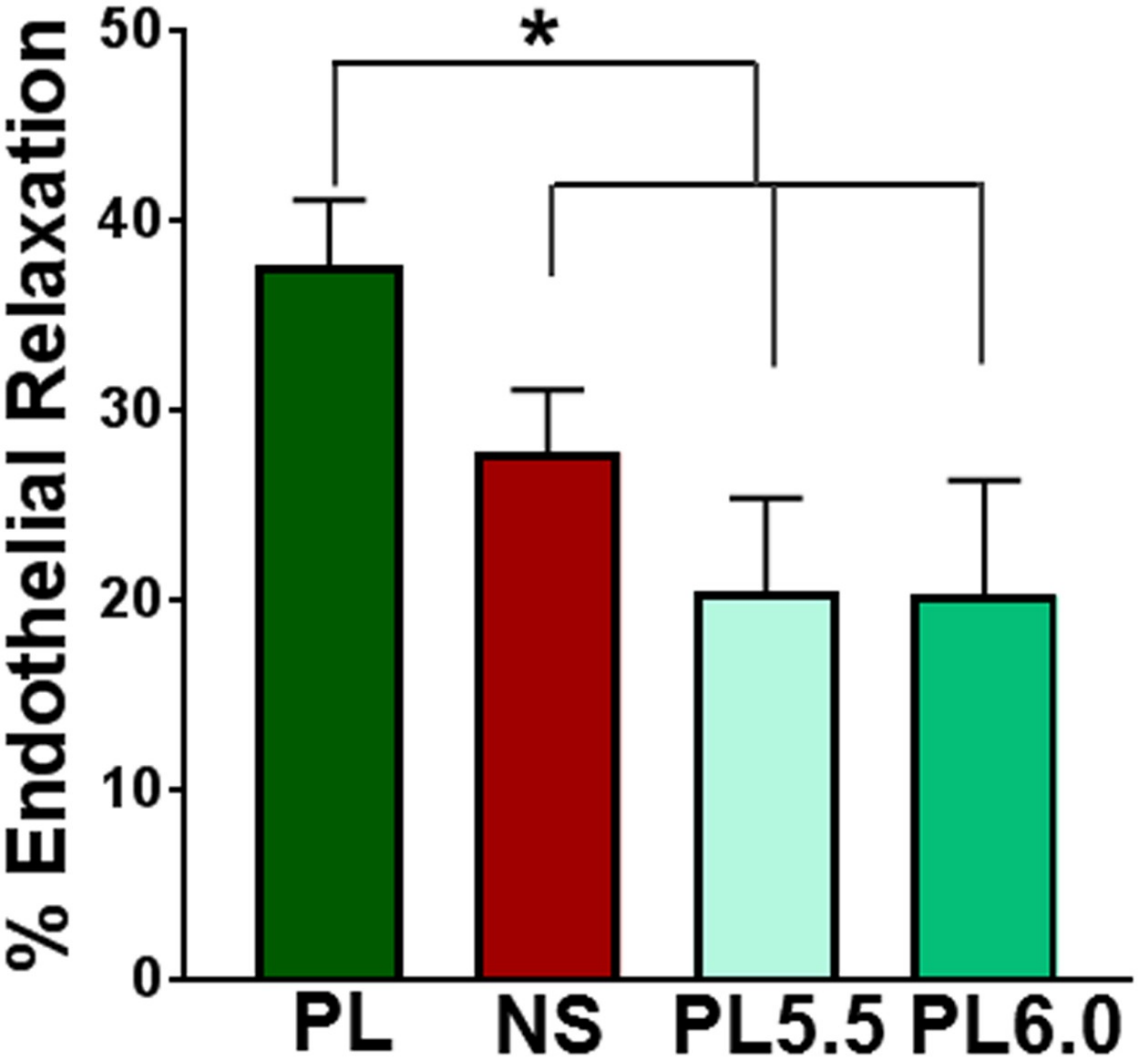
Incubation in Normal Saline (NS) or Plasma-Lyte (PL) rendered acidic leads to decreased endothelial dependent relaxation in intact naïve porcine saphenous veins (PSV). Rings of PSV were incubated in PL; NS; or PL in which the pH had been adjusted to 5.5 or 6.0 for 2 hours. The strips of PSV were then suspended in a muscle bath and pre-contracted with phenylephrine (5 x 10^−7^M) and treated with carbachol (5 x 10^−7^ M, n = 5–7 pigs * p < 0.05).

The pH of PL was 7.0 ± 0.0 (n = 5) and NS was 5.0 ± 0.2 (n = 5 different bags of commercial solutions). To determine if the acidic nature of NS leads to endothelial dysfunction, PSV were harvested prior to hemorrhage and volume overload resuscitation in the porcine model (i.e. naïve tissues) and incubated in the presence of NS, PL, or PL in which the pH had been adjusted to 5.5 or 6.0. Incubation of PSV in NS or PL with pH adjusted to 5.5 or 6.0 led to impaired endothelial function [Fig 5; 37.6 ± 3.5% (PL) vs. 27.8 ± 3.3% (NS), 20.5 ± 4.9% (PL pH 5.5), 20.25 ± 6.0% (PL pH 6.0)].

**Fig 6:**
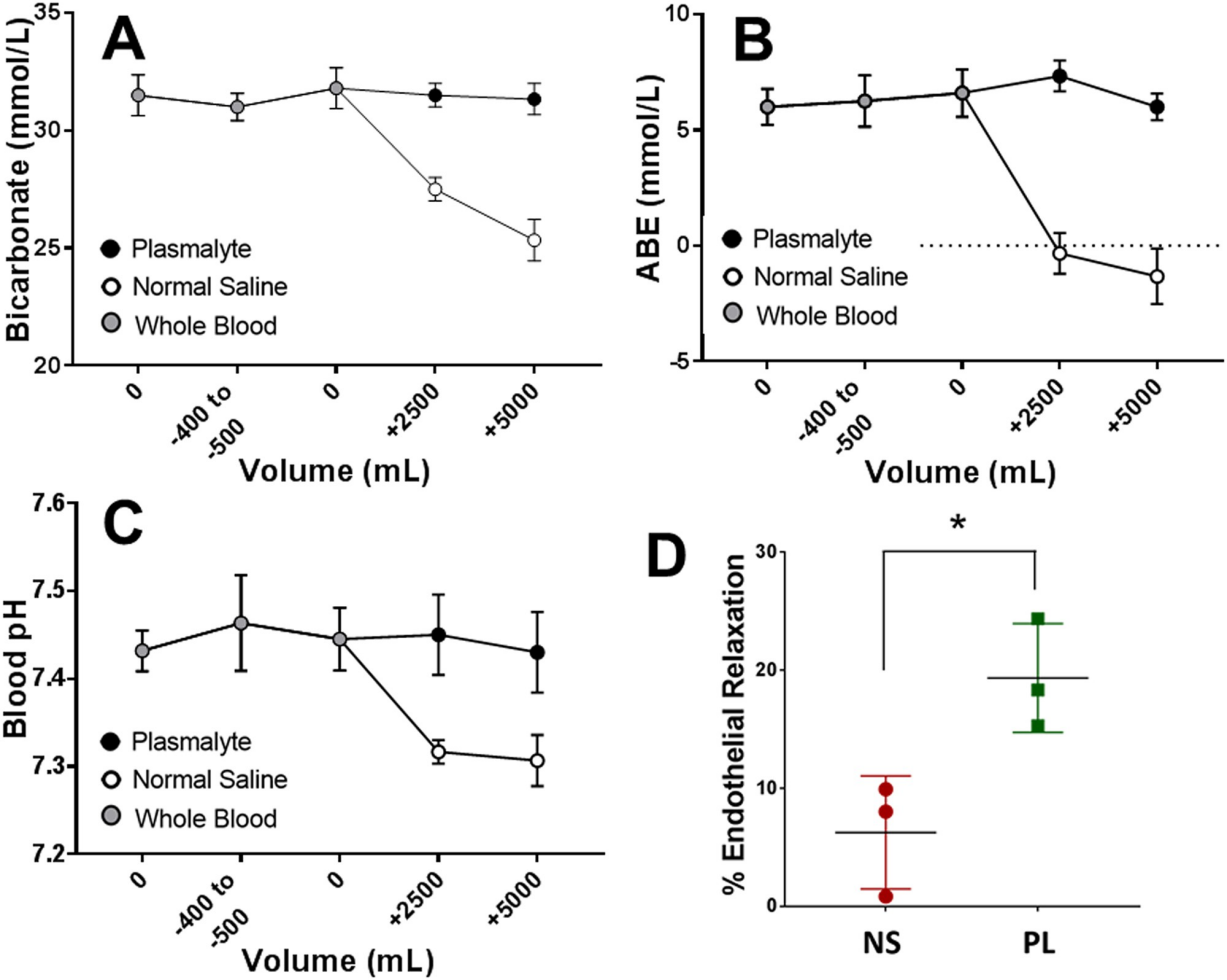
Resuscitation with Normal Saline but not Plasma-Lyte leads to acidosis and endothelial dysfunction in a porcine model. A drop in bicarbonate (**A**, *p < 0.05, n = 3 pigs in each group) arterial base excess (ABE) (**B** *p < 0.05, n = 3 pigs in each group) occurred with 2500 mL and 5000 mL, and a drop in pH occurred with 5000 mL (**C**, * p < 0.05, n = 3 pigs each group) of NS administration. No significant changes occurred during whole blood removal, whole blood return, or PL administration. PSV were atraumatically harvested following resuscitation with NS or PL and suspended in a muscle bath then pre-contracted with phenylephrine (5 x 10^−7^ M) and treated with carbachol (**D**, 5 x 10^−7^ M, n = 3 pigs in each group, * p < 0.05, unpaired t-test).

Blood gas analysis demonstrated that volume overload resuscitation with NS but not PL led to decreases in pH, bicarbonate, and arterial base excess. Decrease in bicarbonate (Fig 6A, p = 0.253; p < 0.05) and arterial base excess (Fig 6B, p < 0.05;) occurred after administration of 2.5L of NS and pH after administration of 5.0L of NS (Fig 6C, p < 0.05). In comparison, volume overload resuscitation with PL did not lead to changes in these systemic acid base parameters.

There were no significant differences in hemodynamic parameters (PCWP or CVP) in pigs resuscitated with NS compared to PL (S5 Fig). Both PCWP and CVP demonstrate a linear relationship with administration of either NS or PL, suggesting that both crystalloid fluid solutions led to similar hemodynamic responses to resuscitation.

To determine if volume overload resuscitation with NS decreased endothelial function *in vivo*, PSV were harvested ***after hemorrhage and volume overload resuscitation***. PSV from pigs that had been resuscitated with NS but not PL displayed impaired endothelial-dependent relaxation (6.2±2.8% vs. 19.3±2.7%); Fig 6D). These data suggest that there is a causal relationship between the acidic nature of NS and endothelial dysfunction, and that *in vivo* volume overload resuscitation with NS but not PL leads to dilutional acidosis and endothelial dysfunction.

## Discussion

It has been well described that storage of HSV in non-buffered NS after harvest and prior to implantation as a vascular bypass graft leads to endothelial dysfunction [3–6, 22]. It has also been demonstrated that resuscitation of patients with NS leads to increased morbidity and mortality [1, 2, 23]. The studies in this report were conducted to determine mechanistically how exposure to NS leads to endothelial dysfunction and the *in vivo* impact of NS resuscitation on endothelial function in a porcine model.

Incubation of cultured HSVEC in NS but not PL, both of which are clinically available electrolyte solutions, led to release of LDH and ATP into the medium and decreased TEER suggesting that exposure to NS leads to increased endothelial permeability and loss of endothelial integrity (Fig 1). Release of cytosolic contents is well known to be associated with membrane injury and tissue damage [24, 25]. NS is nonbuffered and acidic (pH 5.0 ± 0.2). Alterations in the pH of the extracellular microenvironment induces changes in electrical charge of phospholipid head groups in the cell membrane, varying the interfacial interaction and thus the tightness and stability of the lipid bilayer [26, 27]. Thus, it is likely that loss of membrane integrity after exposure to NS is due to the lack of a buffer in NS solution and the acidic pH.

Endothelial-dependent relaxation of intact vascular tissue to acetylcholine is a canonical measure of endothelial function [9]. Incubation of RA in NS led to impaired endothelial-dependent relaxation whereas incubation in PL preserved endothelial function (Fig 2). PSV treated with pH adjusted PL (pH 5.5 or 6.0) led to reduced endothelial-dependent relaxation. This is consistent with previous findings [4] and suggests that it is the acidic nature of NS that leads to endothelial injury.

ATP is present in high concentrations in the cytoplasm (10^−3^ to10^−2^M) and very low concentrations in the extracellular space (10^−9^ to 10^−8^ M; [28, 29]. Treatment of HSVEC with NS led to ATP release (Fig 1) and subfailure overstretch injury of vascular tissue leads to ATP release [11]. In this study, we showed that treatment of RA with ATP or BzATP, a stable synthetic analogue of ATP, led to endothelial dysfunction in buffered PL, further indicating a role of ATP in mediating endothelial injury. Prolonged exposure of cells to high concentrations of extracellular ATP leads to activation of the purinergic receptor, P2X7R [30, 31]. Exposure of RA to NS in the presence of apyrase, which hydrolyzes ATP, or a P2X7R inhibitor, A740003, ameliorated NS-induced endothelial dysfunction (Fig 2A). Similarly, apyrase also abrogated NS-induced endothelial dysfunction in HSV (Fig 2B). Collectively, these data suggest that after loss of endothelial membrane integrity with exposure to NS, ATP release contributes to endothelial dysfunction via activation of P2X7R.

The P2X7R is unusual in that ATP activation of P2X7R leads to further ATP release from the P2X7 receptor itself, due to membrane channels and pores [32]. The associated influx of Ca^2+^ leads to cytolysis and additional release of ATP from intracellular stores. Furthermore, P2X7R-induced apoptotic cell death contributes to additional ATP release into the extracellular milieu [11]. Injury is also associated with increased P2X7R expression [17, 33–35]. This additional ATP may exert paracrine action, activating P2X7R on neighboring cells. Thus, injury and release of ATP, with activation of P2X7R, represents an amplifying feedback process that may potentiate the response to injury.

Injury induced activation of the P2X7R has been associated with activation of the “stress activated” p38 MAPK/MAPKAP kinase 2 (MK2) pathways [36–38]. Treatment of RA or HSV with the p38 MAPK inhibitor SB203580 during NS exposure reduced NS-induced endothelial dysfunction (Fig 2). To confirm that NS exposure leads to p38MAPK activation, HSVECs were incubated in NS or PL, and activation (phosphorylation) of p38MAPK and its downstream substrate MK2 was determined. Exposure of HSVEC to NS but not PL led to increases in the phosphorylation of p38 MAPK and MK2, suggesting that NS leads to activation of stress activated signaling pathways (Fig 3). MK2 activation is associated with increases in phosphorylation of the ribonucleoprotein hnRNPA0 and tristetraprolin (TTP), which stabilize RNA for cytokine production (including IL-1β), resulting in activation of a pro-inflammatory response [14, 15, 39].

ATP treatment of HSVEC led to enhanced IL-1β release that was inhibited by P2X7R inhibitors (oATP or A43) or a p38 MAPK inhibitor, SB (Fig 3). ATP treatment also led to increased expression of the inflammatory marker VCAM in HSVEC that was ameliorated by A43 (Fig 3). Treatment of HSVEC with IL-1β led to increases in phosphorylation of p38 MAPK and MK2 (Fig 4 and S2 Fig). Treatment of RA with IL-1β led to dose dependent decreases in endothelial dependent relaxation (Fig 4) that was ameliorated with p38MAPK inhibition. P2X7R is the most potent activator of the inflammasome [17] and IL-1β can further activate p38 MAPK [20]. Taken together, these data suggest that ATP induced increases in IL-1β may provide a further positive feedback loop for p38 MAPK activation and endothelial dysfunction.

Endothelial-dependent relaxation of vascular smooth muscle is mediated by CCH-induced release of NO. NO is synthesized by nitric oxide synthase (eNOS) from the substrate arginine. Arginase is a hydrolytic enzyme that competes for this substrate and is responsible for conversion of L-arginine into urea and L-ornithine. Treatment with IL-1β did not alter eNOS expression or phosphorylation (S3 Fig) but led to increases in the expression of arginase (Fig 4). Given that arginase can regulate NO production in endothelial cells by competing with eNOS for the substrate L-arginine [40, 41], it is plausible that IL-1β may contribute to endothelial dysfunction by limiting arginine availability for eNOS via increases in arginase activity. Moreover, p38 MAPK activity has previously been associated with increased arginase activity [40] and elevation of arginase has been shown to mediate vascular dysfunction through limiting NO production or availability.

To determine if resuscitation with NS leads to acidosis and endothelial dysfunction in vivo, a porcine model of hemorrhage and volume overload resuscitation with either NS or PL was utilized [7]. There were no differences in hemodynamic responses to resuscitation with either NS or PL (S5 Fig), with both PCWP and CVP demonstrating a linear relationship to volume administered with either PL or NS. However, resuscitation with NS but not PL led to decreased bicarbonate, arterial base excess, and decreased pH (Fig 6A–6C). Decreased bicarbonate levels after volume overload resuscitation with NS suggests that the concurrent acidosis is due to dilution of systemic endogenous buffering capacity rather than a response to the hyperchloremia associated with NS, as has been previously suggested [42]. PSV ***harvested after resuscitation*** with NS but not PL demonstrated impaired endothelial dependent relaxation (Fig 6D), suggesting that clinically relevant doses of NS can lead to acidosis and impaired endothelial function *in vivo*.

Endothelial dysfunction that leads to disturbances of microcirculatory perfusion, organ perfusion defects, and ultimately organ failure after has been attributed to increased permeability of the endothelial monolayer. On the other hand, impaired endothelial-dependent relaxation leads to macrocirculatory perfusion defects and is also referred to as “endothelial dysfunction.” The relationship between these two different forms of endothelial dysfunction is not well known, however, in this study, exposure to NS led to both loss of membrane integrity in cultured endothelial cells and impairment of endothelial-dependent relaxation in intact strips of blood vessels treated with NS ex vivo or strips of intact strips of blood vessels removed after hemorrhage and resuscitation with NS modeled *in vivo*. Taken together, these experimental data suggest mechanisms whereby the acidic nature of non-buffered NS can lead to endothelial dysfunction, both in the form of increased permeability and impaired endothelial-dependent relaxation, and can lead to an amplifying response to injury via activation of inflammatory signaling events.

There are a number of limitations to this study. While release of LDH and ATP after exposure of tissues and cells to acidic solutions is consistent with loss of membrane integrity, the specific effects of acidosis on the phospholipid bilayer have not been clearly elucidated. The mechanistic links and long-term consequences of injury-induced ATP release and P2X7R activation and potential amelioration of the cascade of inflammatory induced responses with P2X7R antagonists requires further evaluation in *in vivo* models. Further limitations of this study include the use of cultured primary cells to determine activation and expression levels of p38MAPK/MK2, IL-1β, VCAM, and arginase. However, cultured HSVEC allows for cell-type specific responses that are not feasible with intact vascular tissues. Additionally, although we demonstrate activation of arginase expression with IL-1β, which may limit NO substrate availability, actual NO and L-arginine levels were not determined. In the porcine model, over resuscitation was performed to the level of decreased cardiac output. Healthy pigs likely require a larger amount of volume resuscitation before decreased cardiac output occurs than injured humans, but this represents a physiologic endpoint based on the Starling curve. The pigs used in this study were healthy and the responses observed may not reflect human responses under pathologic conditions.

## Conclusions

**Fig 7:**
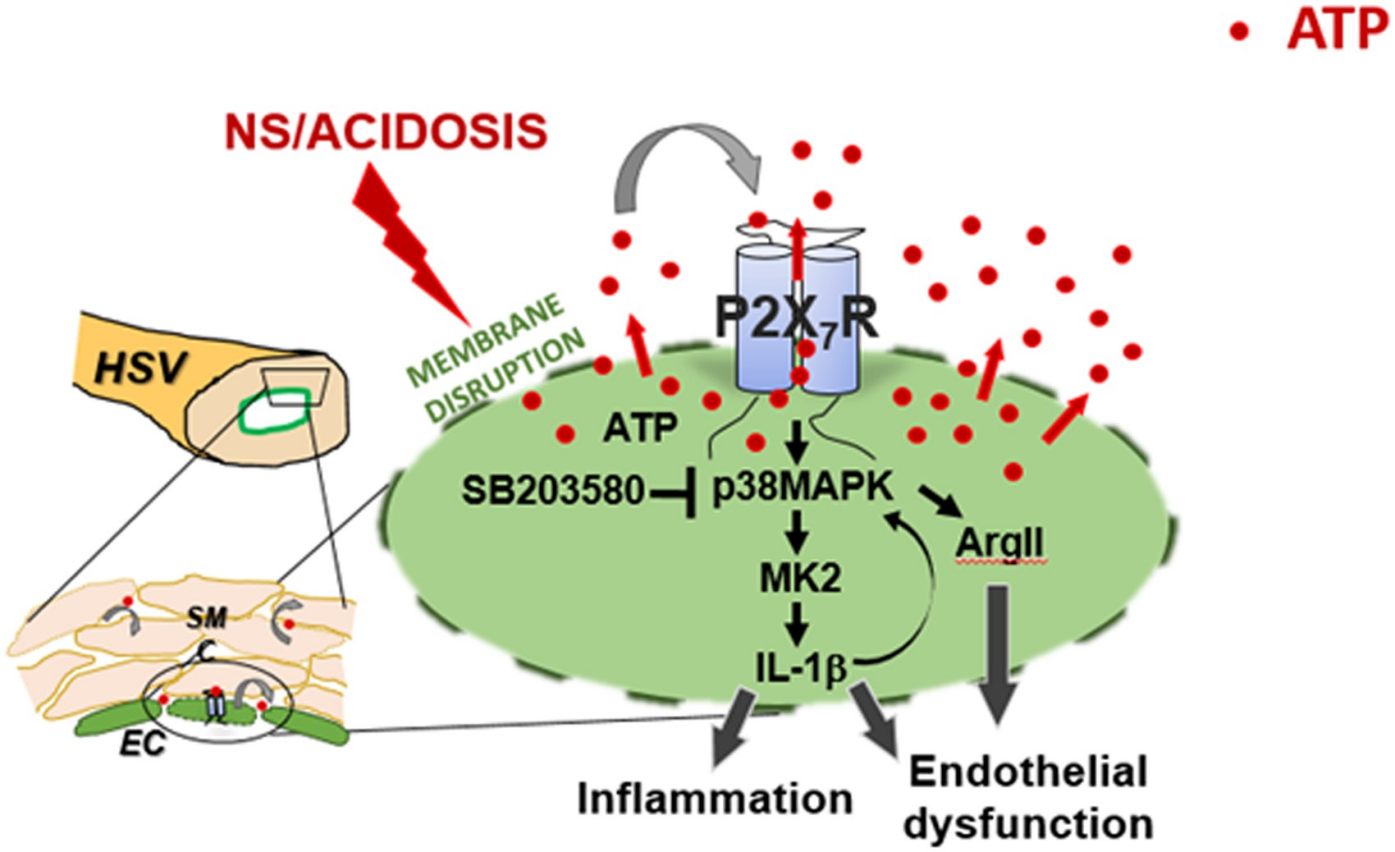
Model of molecular mechanisms of vascular injury to endothelium by ATP. Injury to vascular tissues (including storage of vein graft in Normal Saline) leads to membrane disruption and ATP release. Extracellular ATP activates the P2X_7_R/p38MAPK pathway, leading to IL-1β production and inflammation. Activation of P2X_7_R leads to increased extracellular ATP via release from P2X_7_R channels/pores. Prolonged exposure to IL-1β leads to increased arginase II expression and activation of P38MAPK, which contributes to endothelial dysfunction and amplification of the response to injury.

Taken together, these data suggest that exposure of the endothelium to non-buffered, acidic Normal Saline solution leads to loss of endothelial cell membrane integrity, release of ATP, activation of the P2X7R/p38MAPK/MK2 signaling axis. This leads to potentiation of the injury response through amplification of ATP release and P2X7R activation as well as cytokine (IL-1β) release and amplification of p38 MAPK activation and increases in arginase activity. We demonstrate that the physiologic result of these perturbations is endothelial dysfunction, observed both experimentally and *in vivo*. These data pose a potential direct link between acidotic vascular injury, inflammatory responses, endothelial dysfunction and potentiation of the injury response (Fig 7). These data also suggest that the acidotic milieu associated with the use of Normal Saline contributes to endothelial dysfunction in the form of both increased endothelial permeability and impaired endothelial-dependent relaxation. These experimental results provide mechanistic support to mounting clinical data which suggest that balanced buffered crystalloid solutions are preferred for fluid resuscitation.

## Supporting information

S1 Fig Change in TEER in HSVEC. HSVEC were incubated in basal medium (Ctrl), Normal Saline (NS) or Plasma-Lyte (PL) and TEER was measured at 0.5, 120, 24, 48 and 72 hours. Control medium was added to all cells after 2 h of incubation with NS. Change in TEER is plotted. *, p < 0.05, n = 4, in triplicates from different passage cells.
(TIF)

S2 Fig IL-1β leads to activation of p38MAPK and MK2 in HSVEC. Cells were either untreated (control, Ctrl) or treated with IL1-β (10 ng/ml) for 10, 30, 60, 180 and 360 min. Cell lysates were prepared and immunoblotted for phospho- and total p38MAPK (A) and MK2 (B). Top, representative immunoblots; bottom: cumulative data of relative phosphorylation compared to untreated cells. *p<0.05, n = 4–5.
(TIF)

S3 Fig Treatment with interleukin-1β (IL-1β) does not change endothelial nitric oxide synthase (eNOS) expression or phosphorylation in human saphenous vein endothelial cells (HSVEC). Cells were either untreated (control) or treated with IL-1β (10 ng/ml) for 3, 6 and 24hours. Cell lysates were prepared and immunoblotted for p-eNOS and eNOS and normalized to GAPDH (glyceraldehyde-3-phosphate dehydrogenase) expression. Top, representative immunoblots; bottom: cumulative data of phosphorylation of eNOS and protein levels compared to untreated cells (*p≤0.05, n.s., not significant, n = 4–7).
(TIF)

S4 Fig Time course of endothelial nitric oxide synthase expression and phosphorylation in the presence of IL-1β in HSVEC. Cells were either untreated (control) or treated with IL-1β (10 ng/ml) for 10, 30, 60, 180 and 360 min. Cell lysates were prepared and immunoblotted for phospho- and total eNOS. Top, representative immunoblots; middle: cumulative data of relative eNOS levels; and bottom: cumulative data of relative eNOS phosphorylation compared to untreated cells. ns, n = 4.
(TIF)

S5 Fig Hemodynamic responses to hemorrhage and volume overload resuscitation to NS or PL in a porcine model. There was a linear relationship between central venous pressure (CVP) and volume with both Normal Saline (**A**, r = 0.60, n = 42) or Plasma-Lyte (**B**, r = 0.70, p<0.05, n = 43). There was a linear relationship between pulmonary capillary wedge pressure (PCWP) and volume to resuscitation with Normal Saline (**C**, r = 0.61, p<0.05, n = 47) or Plasma-Lyte (**D**, r = 0.81, p<0.05, n = 43).
(TIF)
