# Supplementary figures and images for: Normal Saline solutions cause endothelial dysfunction through loss of membrane integrity, ATP release, and inflammatory responses mediated by P2X7R/p38 MAPK/MK2 signaling pathways
Source: PLoS One. 2019 Aug 14;14(8):e0220893. doi: 10.1371/journal.pone.0220893 (PMC6693757; doi:10.1371/journal.pone.0220893)

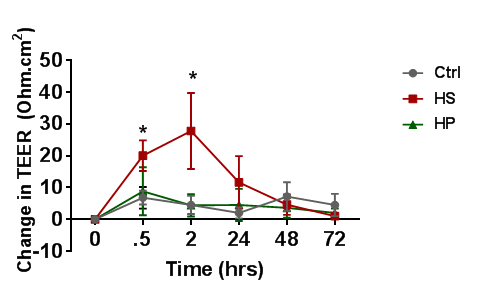

Supplement: S1 Fig — HSVEC were incubated in basal medium (Ctrl), Normal Saline (NS) or Plasma-Lyte (PL) and TEER was measured at 0.5, 120, 24, 48 and 72 hours. Control medium was added to all cells after 2 h of incubation with NS. Change in TEER is plotted. *, p < 0.05, n = 4, in triplicates from different passage cells. (TIF) [file pone.0220893.s001.tif]

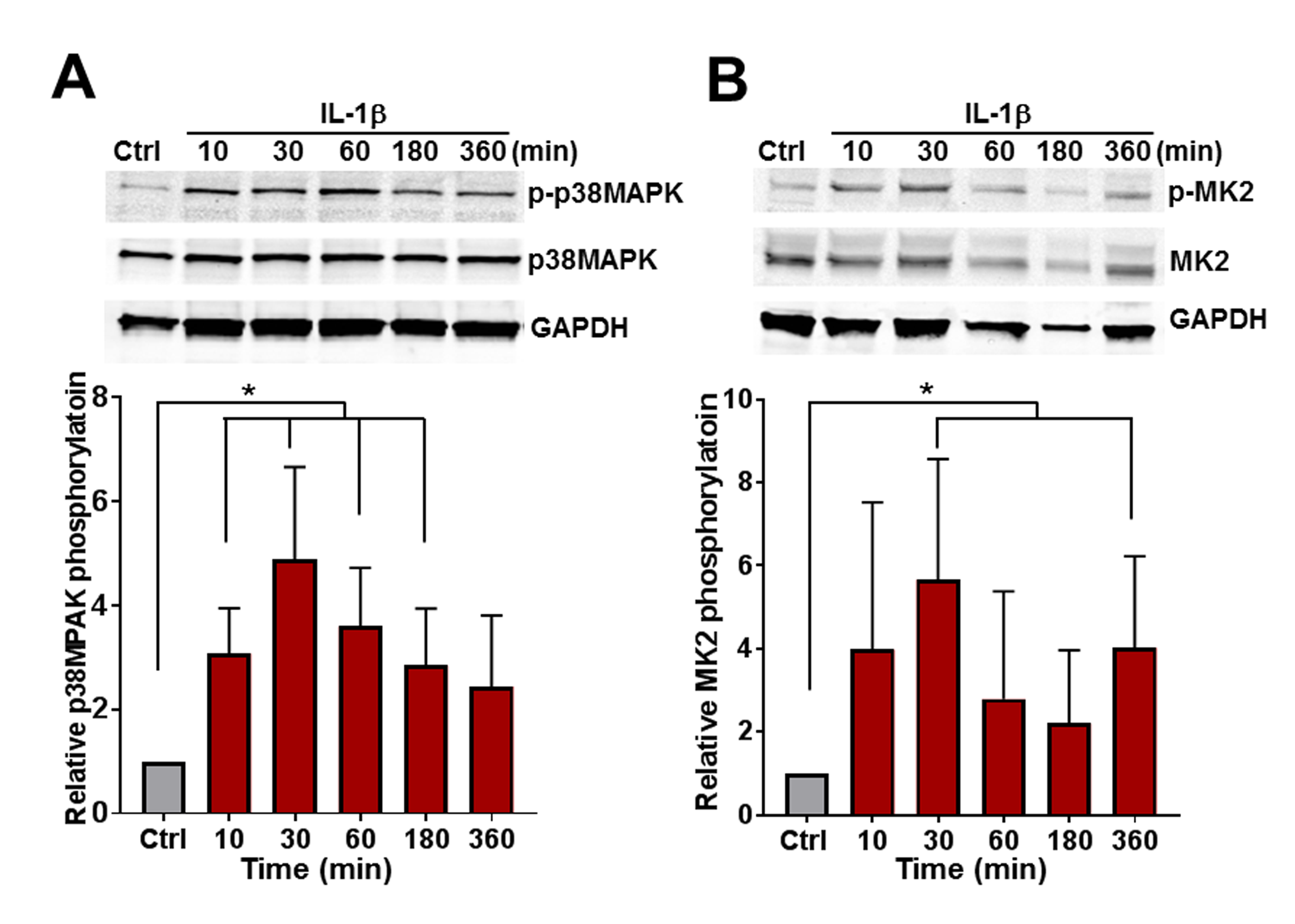

Supplement: S2 Fig — Cells were either untreated (control, Ctrl) or treated with IL1-β (10 ng/ml) for 10, 30, 60, 180 and 360 min. Cell lysates were prepared and immunoblotted for phospho- and total p38MAPK (A) and MK2 (B). Top, representative immunoblots; bottom: cumulative data of relative phosphorylation compared to untreated cells. *p<0.05, n = 4–5. (TIF) [file pone.0220893.s002.tif]

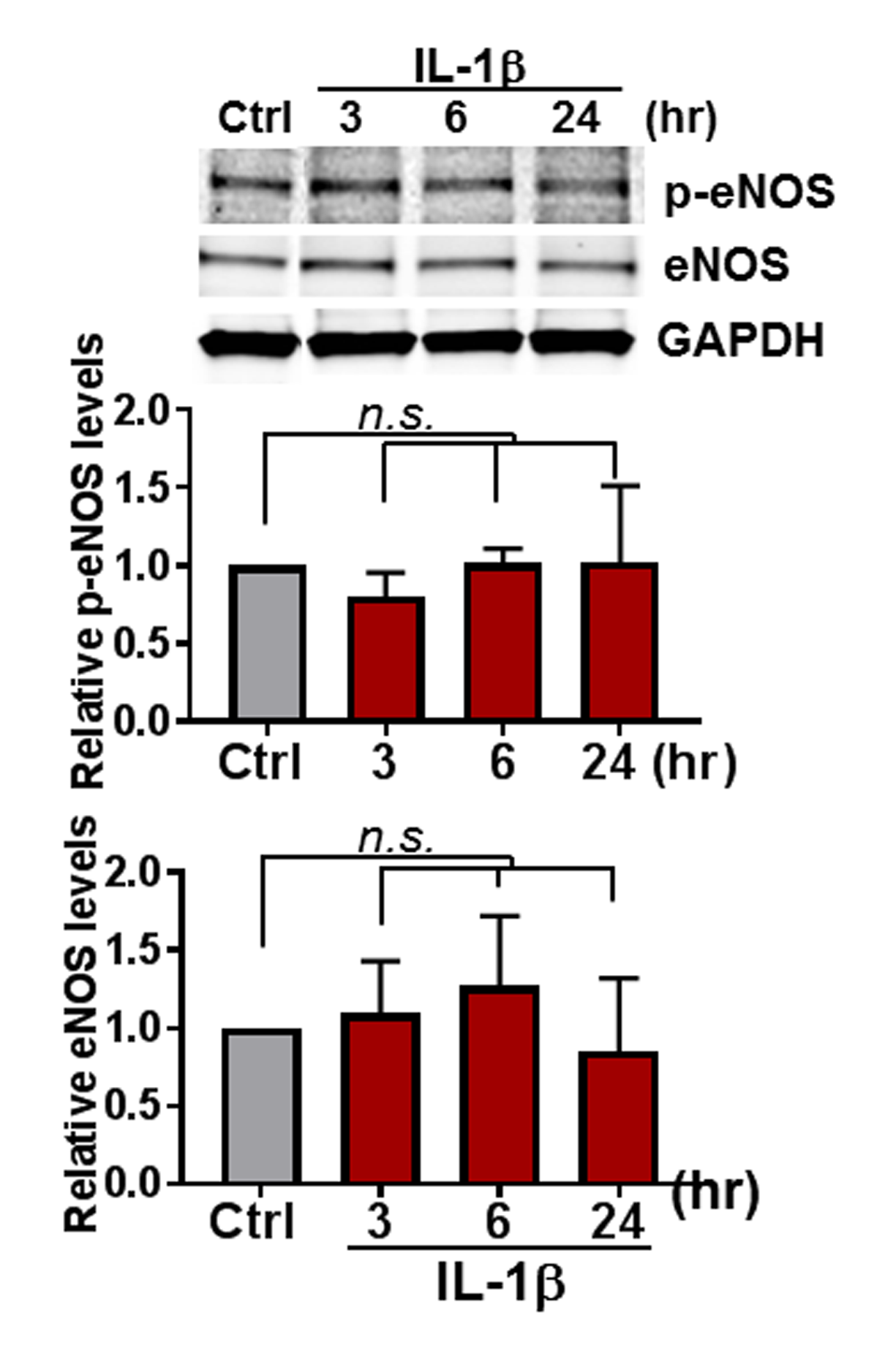

Supplement: S3 Fig — Cells were either untreated (control) or treated with IL-1β (10 ng/ml) for 3, 6 and 24hours. Cell lysates were prepared and immunoblotted for p-eNOS and eNOS and normalized to GAPDH (glyceraldehyde-3-phosphate dehydrogenase) expression. Top, representative immunoblots; bottom: cumulative data of phosphorylation of eNOS and protein levels compared to untreated cells (*p≤0.05, n.s., not significant, n = 4–7). (TIF) [file pone.0220893.s003.tif]

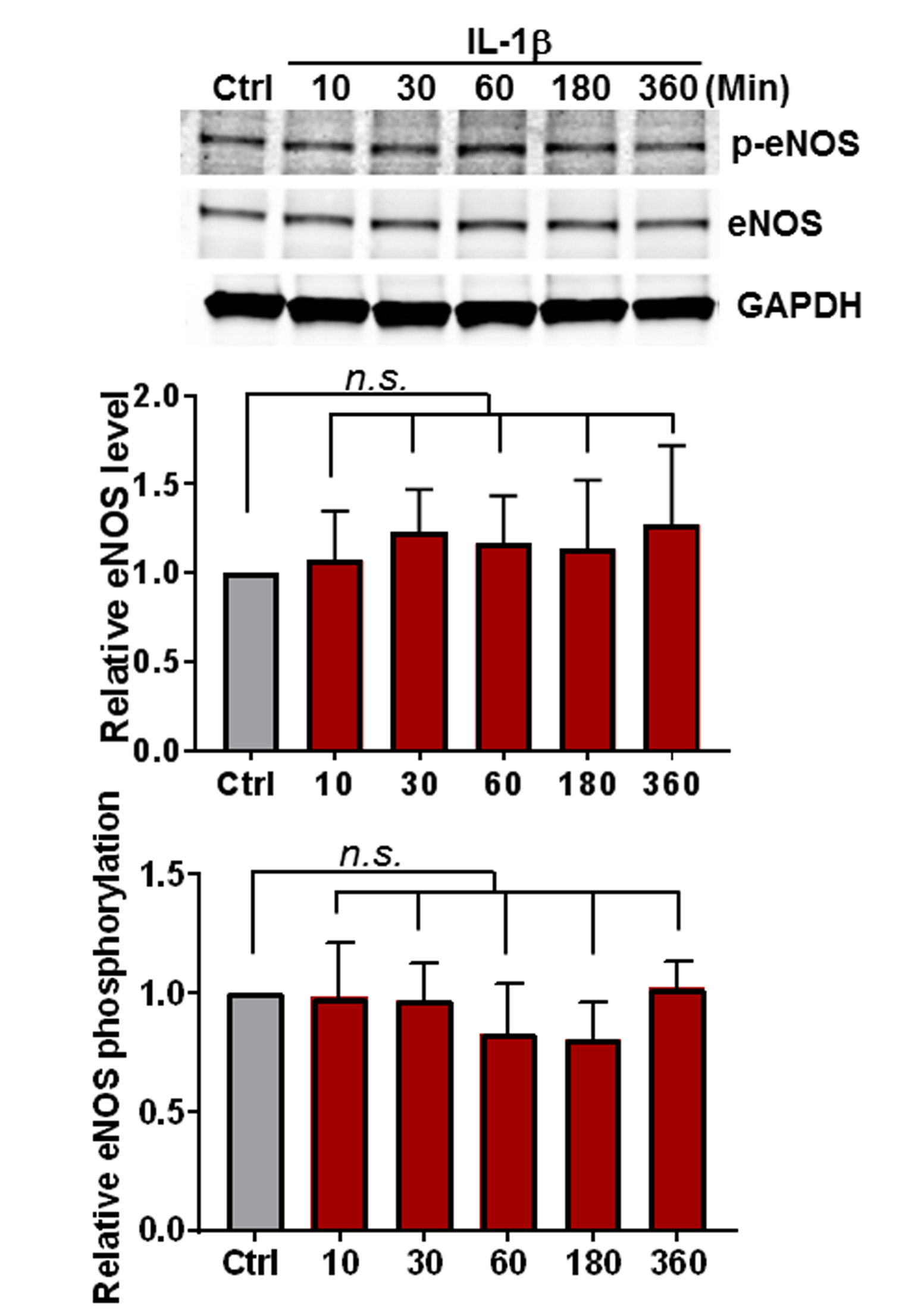

Supplement: S4 Fig — Cells were either untreated (control) or treated with IL-1β (10 ng/ml) for 10, 30, 60, 180 and 360 min. Cell lysates were prepared and immunoblotted for phospho- and total eNOS. Top, representative immunoblots; middle: cumulative data of relative eNOS levels; and bottom: cumulative data of relative eNOS phosphorylation compared to untreated cells. ns, n = 4. (TIF) [file pone.0220893.s004.tif]

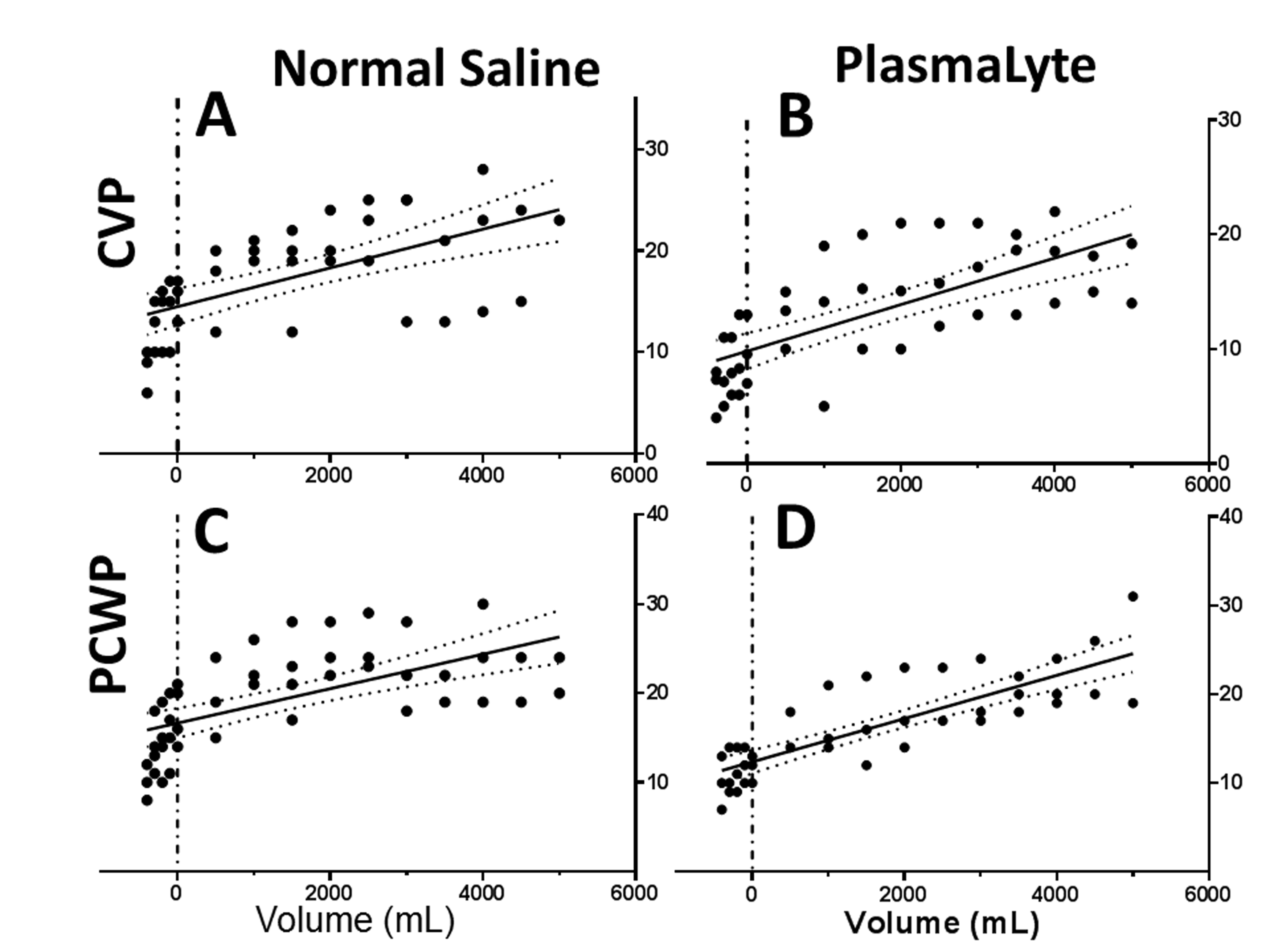

Supplement: S5 Fig — There was a linear relationship between central venous pressure (CVP) and volume with both Normal Saline (A, r = 0.60, n = 42) or Plasma-Lyte (B, r = 0.70, p<0.05, n = 43). There was a linear relationship between pulmonary capillary wedge pressure (PCWP) and volume to resuscitation with Normal Saline (C, r = 0.61, p<0.05, n = 47) or Plasma-Lyte (D, r = 0.81, p<0.05, n = 43). (TIF) [file pone.0220893.s005.tif]
